# Projection of Young-Old and Old-Old with Functional Disability: Does Accounting for the Changing Educational Composition of the Elderly Population Make a Difference?

**DOI:** 10.1371/journal.pone.0126471

**Published:** 2015-05-14

**Authors:** John P. Ansah, Rahul Malhotra, Nicola Lew, Chi-Tsun Chiu, Angelique Chan, Steffen Bayer, David B. Matchar

**Affiliations:** 1 Signature Program in Health Services and Systems Research, Duke-NUS Graduate Medical School, Singapore, Singapore; 2 Department of Sociology, National University of Singapore, Singapore, Singapore; 3 Department of Medicine, Duke University Medical Center, Durham, North Carolina, United States of America; University of Padova, ITALY

## Abstract

This study compares projections, up to year 2040, of young-old (aged 60-79) and old-old (aged 80+) with functional disability in Singapore with and without accounting for the changing educational composition of the Singaporean elderly. Two multi-state population models, with and without accounting for educational composition respectively, were developed, parameterized with age-gender-(education)-specific transition probabilities (between active, functional disability and death states) estimated from two waves (2009 and 2011) of a nationally representative survey of community-dwelling Singaporeans aged ≥60 years (N=4,990). Probabilistic sensitivity analysis with the bootstrap method was used to obtain the 95% confidence interval of the transition probabilities. Not accounting for educational composition overestimated the young-old with functional disability by 65 percent and underestimated the old-old by 20 percent in 2040. Accounting for educational composition, the proportion of old-old with functional disability increased from 40.8 percent in 2000 to 64.4 percent by 2040; not accounting for educational composition, the proportion in 2040 was 49.4 percent. Since the health profiles, and hence care needs, of the old-old differ from those of the young-old, health care service utilization and expenditure and the demand for formal and informal caregiving will be affected, impacting health and long-term care policy.

## Introduction

Aging is an issue that will impact population demographics and health profiles everywhere. Between 2000 and 2040, the number of people at least 60 years of age worldwide is projected to grow from 610 million to more than 2 billion [1]. Medical advances have improved health and delayed mortality, resulting in a shift in population age structures globally. In part due to these reasons and significant developments in overall living standards, the Southeast Asian city-state Singapore has transitioned from a young population with high fertility and mortality rates in the 1980s to an aging population characterized by low mortality and below-replacement level fertility rates [2–4]. This demographic shift has also largely been fueled by the aging post-war baby boomer generation. In Singapore, the proportion of elderly aged 60 years and older is expected to rise from 16 percent of the population in 2014 to over 30 percent of the population by 2040 [1]. Within this cohort, the group aged 80+ is anticipated to increase more than 4 times, from 121,800 in 2014 to 567,500 in 2040 [1]. This rapid growth of the elderly population is a source of concern due to the health implications of aging.

While people are increasingly avoiding fatal events, they are often not avoiding the physiological changes associated with aging and the accumulation of chronic conditions such as functional disability [5–10]. In 2001, the WHO adopted the International Classification of Functioning, Disability and Health (ICF) to measure health and disability more broadly. In this paper, however, we look specifically at functional disability, which we have operationally defined as difficulty performing one or more activities of daily living (ADLs) or instrumental ADLs (IADLs). Functional disability is most common among the elderly [11], increasing their care needs, impacting family and other caregivers, and affecting health care utilization and expenditure. Thus, functional disability is a significant issue that accompanies population aging and merits great attention.

Since the 1980s, the elderly population in many countries has been substantial enough to warrant a sub-division of the cohort comprising individuals above the age of 60 [12–15], helping researchers identify the heterogeneity of the elderly population. Researchers have acknowledged the variability among those aged 60 and above and conducted multiple studies attempting to determine the characteristics of the burgeoning old-old (aged 80+ years) cohort [16–19]; some have even suggested a delay of the traditional cut-off point of 60 or 65 that demarcates entry into old age in light of the different health profiles of those aged 70, 78, and 85 [20]. Age-specific variations in mortality rates above 85 [21], as well as elevated risks of developing health problems like depression [22], dementia [23–25] and disability [20, 24] among the old-old have also supported the further delineation of age groups among those above 60. In this study, we too distinguish two cohorts of the elderly, the “old-old” and the “young-old,” to describe those aged 80+ and those aged 60–79 years respectively.

Aging alone could lead to undesirable consequences from a policymaker’s perspective such as rising dependency, greater health care utilization [26] and escalating health care costs [27–29]. Additionally, numerous studies have demonstrated that the old-old have higher rates of health services utilization for both acute care [20, 30, 31] and long-term care [32, 33] than the young-old. A rise in functional disability could exacerbate these problems. Elderly with functional disability are associated with greater formal long-term care use [34, 35] and acute care utilization [36], and may result in growing health care expenditures [37]. Primary caregivers of those with severe disability also display elevated risks of high stress and depression [38], placing greater strain on the health care system. Furthermore, there could be unanticipated effects on other sectors: research shows that caregivers of elderly with functional disability may choose to give care full-time [39], potentially decreasing participation in the labor market. The importance of studying functional disability is amplified when we consider that functional disability is a crucial determinant of service needs. The ability to perform ADLs and IADLs is necessary for independent living and is thus a good indicator of the future need for caregivers, home-based and community services, as well as nursing homes, which are all relevant to policymaking. Currently, disability measures are used to assess individuals’ qualification for care programs and aid schemes. In Singapore, disabled Singaporeans are eligible to claim benefits under the ElderShield disability insurance scheme [40]. Meanwhile, in Taiwan, disabled citizens over 50 years of age are eligible to receive services such as home rehabilitation and nursing under the national 10-year long-term care plan [41]. Hence, functional disability projections are useful tools for policymakers to assess the elderly population’s health and social care needs and mitigate the negative consequences of aging and disability.

In the past few decades, studies have shown that socioeconomic factors, particularly education, are strong predictors of mortality and morbidity [42]. A higher level of education is associated with lower levels of disability, morbidity and mortality [42, 43]. The relationship between education and disability has been supported in a number of populations using different methodologies, and measures such as the ability to perform ADLs and IADLs, cognitive ability and mobility status (needing help climbing stairs or walking half a mile), though not consistently [44]. Freedman and Martin [45] provided early evidence supporting the effect of education on declines in functional limitations among elderly Americans; of eight demographic and socioeconomic variables considered (education, age, sex, race, ethnicity, marital status, financial assets, and region), they found that education was the most important in explaining the declines in functional limitations. Sulander and colleagues [46] made a similar finding among Finns aged 65–84 years: those with a higher education had significantly better functional ability (lower prevalence of ADL limitations) than those with a lower education, even after adjusting for age, survey period, chronic diseases and depressive symptoms.

In making functional disability projections, the differential effects of education on disability incidence, recovery and mortality are particularly relevant. Multiple studies have associated a lower level of education with higher disability incidence [47–51], though this association may depend somewhat on the disability measure used. For instance, Jagger and colleagues [52] found that having 9 years of full-time education or less was associated with higher incidence of mobility disability in both men and women, but was only associated with higher incidence of ADL disability in women. While several studies have found no relationship between education and recovery from disability [48–51], Jagger and colleagues [52] found that those with 9 years of education or less had a lower recovery of mobility disability. Meanwhile, researchers examining education and mortality in those with disability found no association between the two [47, 48, 52]. Nonetheless, the studies examining education and incidence and recovery suggest that failing to account for education could affect functional disability projections.

Similar studies conducted in Asia suggest that the strong association between education and disability incidence observed in Western societies may not translate similarly in an Asian setting. A study conducted in 2012 among elderly Japanese found statistically significant differences by education in disability incidence but not recovery from disability (though this could be due to the small sample sizes for mortality and recovery states and the transitions therein) [47]. An older study conducted in 1998 in Taiwan similarly only found an association between educational levels and disability incidence [53]. However, studies published in 2007 in Indonesia that examine the relationship between education and disability using functional health status, active life expectancy, or functional transitions as a measure have not found robust associations between education and disability [54, 55]. While Kaneda and Zimmer cite the low education rates among the elderly in Indonesia as a potential reason for the weak association [55], the variation in education and disability trends that countries in Asia display highlights the need for further research in this area not only in Asia but also in individual countries like Singapore.

In Singapore and many other countries, as the educational composition of the future elderly population is changing rapidly, its effect on disability projections could be substantial. Similarly, countries like China, South Korea and Thailand have undergone fundamental changes in the past few decades; their governments have placed a strong emphasis on education, affecting the educational makeup of their populations. However, most current disability projections fail to account for the changing educational composition of the future elderly population. Disability projections have been made to gauge the future long-term care need in the U.S. [56], U.K. [57] and Australia [58], as well as to determine the costs of caring for the population of disabled [59, 60], but, to our knowledge, only one study thus far makes functional disability projections taking changing educational composition of the population into account. In this study, Samir & Lentzner [43] used sample data from 70 countries that participated in the World Health Survey to project levels of disability to 2050 and reported that the prevalence of disability was consistently lower when they accounted for changing educational composition of the population in their projections.

Against this backdrop, projecting functional disability among elderly Singaporeans provides an ideal setting for considering the practical impact of educational composition of the elderly population. This study aimed to compare projections, up to year 2040, of young-old (aged 60–79) and old-old (aged 80+) with functional disability in Singapore with and without accounting for the changing educational composition of the Singapore elderly population and achieved this aim.

## Materials and Methods

To determine if the future number of Singaporean elderly with functional disability would differ with and without accounting for a changing educational composition, two dynamic multi-state population models—one not accounting for educational composition and the other accounting for educational composition in disability transition probabilities, fertility rates, and mortality rates—were developed. The models required standard demographic data as well as estimates of the following transition probabilities: from active to functional disability, from functional disability to active, from active to death and functional disability to death. Demographic data was obtained from the Department of Statistics Singapore (S1–S5 Files). The transition probabilities and retention probabilities (probability of remaining in the same state) were estimated from two waves of a nationally representative survey of community-dwelling Singaporeans aged ≥ 60 years (N = 4,990)—Wave 1, referred to as the Social Isolation, Health and Lifestyles Survey (SIHLS), conducted in 2009, and Wave 2, referred to as the Panel on Health and Aging of Singaporean Elderly (PHASE), conducted in 2011. Sampling methodology and survey details of the SIHLS [61] is available from the literature as cited and more details on each of these components are given below.

### Dynamic multi-state population model

Two dynamic multi-state population models (i.e., with and without accounting for educational composition) were constructed using Vensim DSS (Ventana Inc). The dynamic multi-state population models were continuous time compartment models with explicit population stocks.

**Fig 1:**
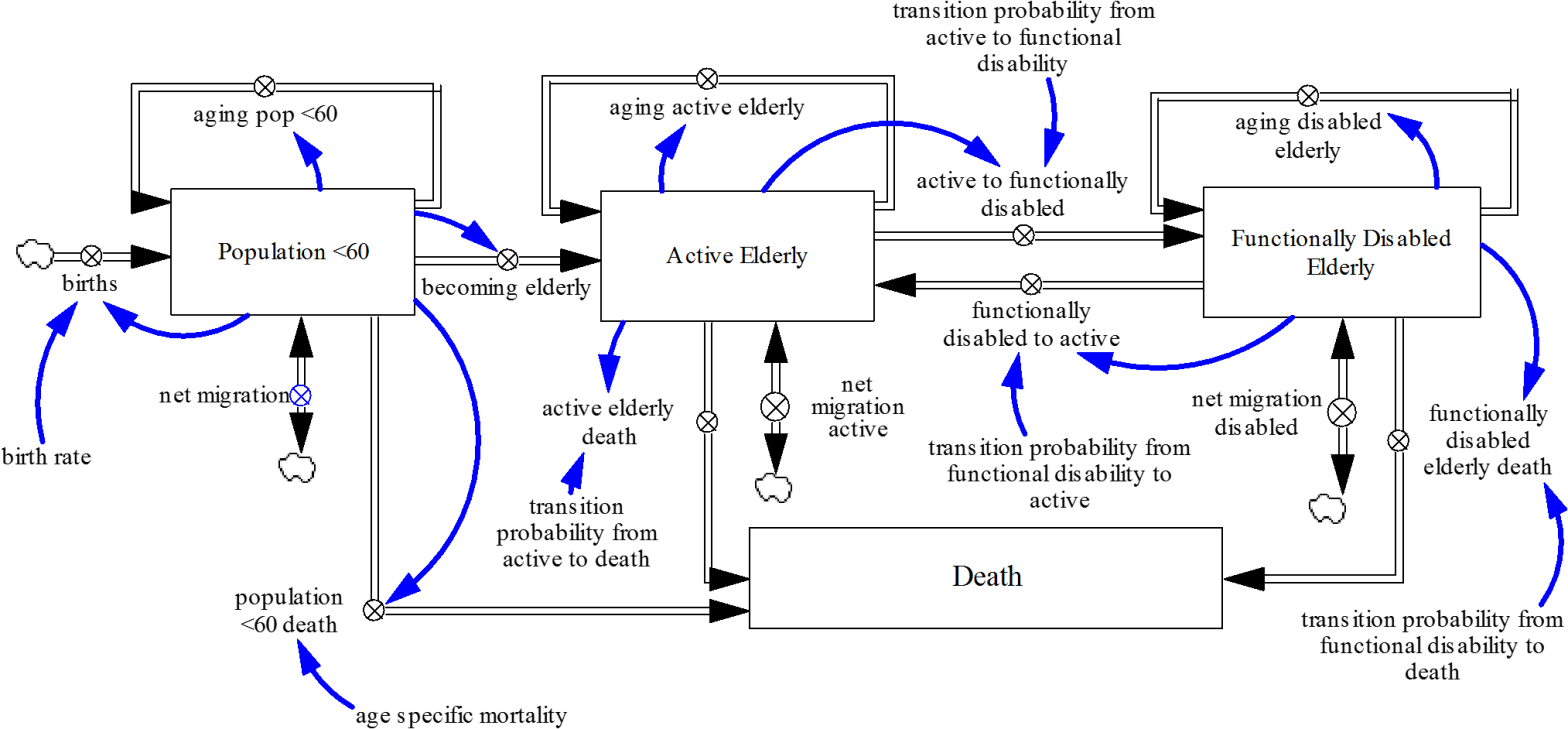
Dynamic Multi-state Population Model Not Accounting for Education. Population model with fertility rates, mortality rates and disability transition probabilities that are not education-specific.

Fig 1 depicts the structure of the model not accounting for educational composition.

**Fig 2:**
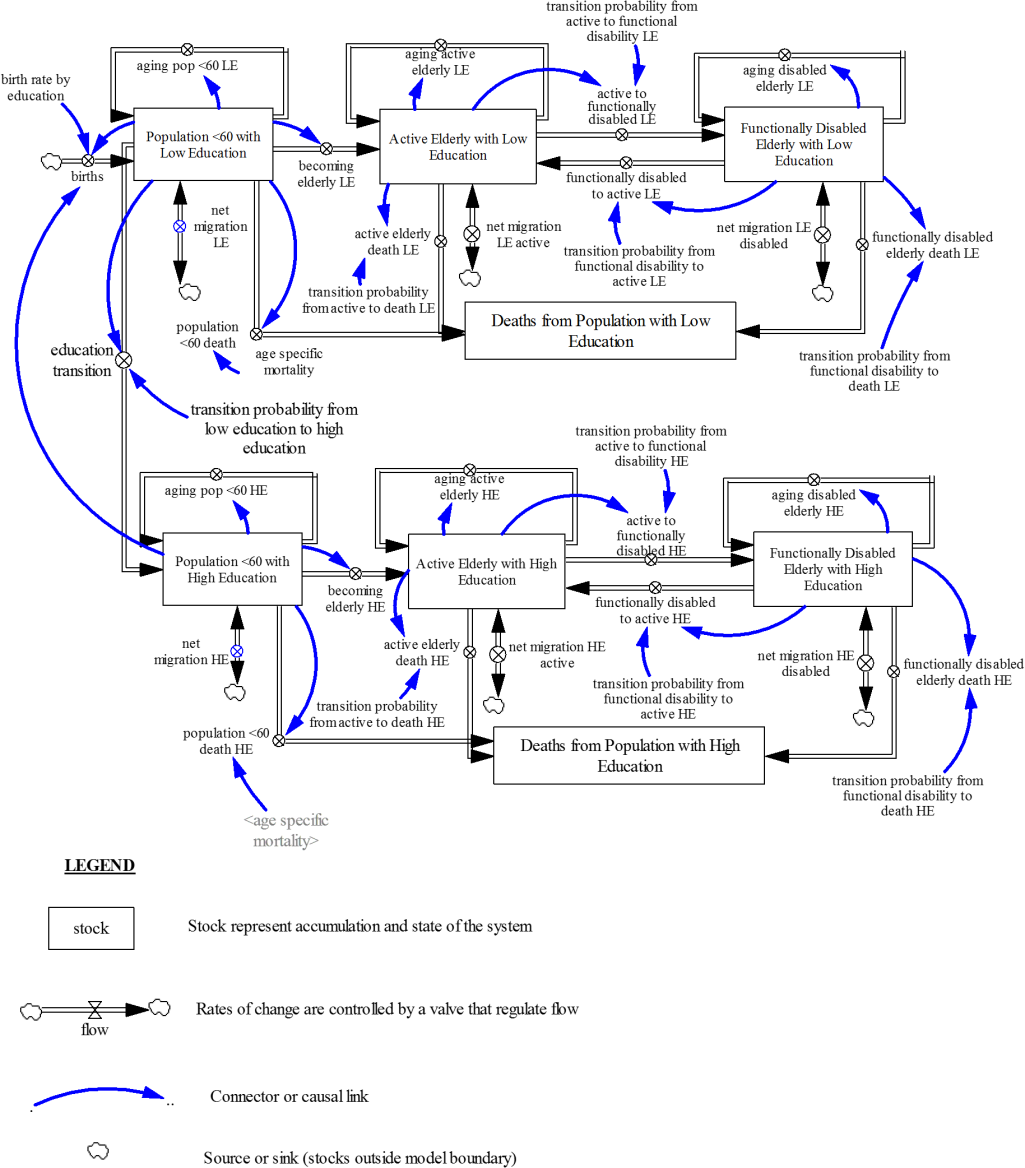
Dynamic Multi-state Population Model Accounting for Education. Population model with education-specific fertility rates, mortality rates and disability transition probabilities.

Fig 2 depicts the structure of the model accounting for educational composition.

In the model that does not account for educational composition, the Singapore population (made up of Singapore citizens and permanent residents) was divided into three groups as seen in Fig 1: the population under 60, active (functionally able) elderly, and functionally disabled elderly. Within each group, the population was further sub-divided by age (single age cohorts from age 0—age 59 years for the under 60 group and single age cohorts from age 60-age 100 and older for the active and functionally disabled elderly groups) and gender. The population under 60 increases via births and immigration and decreases via deaths, emigration and becoming elderly (aging from 59 to 60). Births were calculated based on the female reproductive age cohort (aged 15–44) and their corresponding fertility rates [62] while deaths were calculated using mortality rates for each age cohort obtained from life tables [62]. Emigration was estimated by calibration [63]. The aging process ensures that at the end of every year, the surviving population in each age cohort transitions to the subsequent cohort except for the final age cohort (aged 100 and older). The model configuration allows elderly Singaporeans to transition between the active and functional disability states, or flow out from either state as they die or emigrate. Disability transition probability estimates used here were not specific for education.

In Singapore, individuals typically attend pre-school from the ages of 4–6, primary school from 7–12, secondary school from 13–16, post-secondary school from 17–19, and university from 20–23 [64]. In the model that accounts for education, the fertility rates, mortality rates and disability transition probabilities were education-specific as seen in Fig 2. Between ages 15 to 19, individuals could transition from low education (primary school education or less) to high education (secondary school education or higher), with this transition probability from low education to high education decreasing from 15 to 19 in line with past trends observed in the Singapore education system [65]. This demarcation was deemed appropriate due to low educational attainment levels of the current cohort of elderly, who did not benefit from Singapore’s subsequent push for greater education. The education transition assumes that changes in education by age and gender are uni-directional i.e. over time, people can only remain at their current educational level or move to a higher educational level and cannot move backward. It must be noted that no one is born into the high education population cohort because the transition into this cohort is assumed to occur between ages 15 to 19. Hence, all individuals below this age range are in the low education population cohort. Similar to the model described above, the low education and high education groups were each divided into three groups: the population under 60, active elderly and functionally disabled elderly. Within each group, the population was further sub-divided by age (single age cohorts from age 0—age 59 years for the under 60 group and single age cohorts from age 60-age 100 and older for the active and functionally disabled elderly groups) and gender.

### Assumptions

1. Fertility rate
  - The multi-state population model not accounting for educational composition used total fertility rates of Singapore from 2000 to 2013 as provided by the Department of Statistics Singapore to populate the model (S3 File). The fertility rate from 2013 to 2040 was assumed to remain constant.
  - The multi-state population model accounting for educational composition used fertility rates by education from 2000 to 2013 as provided by the Department of Statistics Singapore to populate the model (S3 File). The fertility rates by education from 2013 to 2040 were assumed to remain constant.
2. Mortality rate
  - The multi-state population model not accounting for educational composition applied age-specific mortality rates from 2000 to 2013 provided by the Department of Statistics Singapore (S1–S2 Files) to the population below 60 years of age. Mortality rates for those under 60 were assumed to remain constant from 2013 to 2040. For the population above 60, the mortality rates were estimated from SIHLS and PHASE. Likely changes in mortality rates for those above 60 were addressed using bootstrap estimates.
  - The multi-state population model accounting for educational attainment applied age-specific mortality rates not disaggregated by education from 2000 to 2013 to the population below 60 years of age as mortality rates by education were not provided by the Department of Statistics Singapore. Hence, it was assumed that there were no educational differences in mortality for individuals below 60 years of age. Mortality rates for those under 60 were assumed to remain constant from 2000 to 2013. For individuals 60 years or older, mortality rates by education were estimated from SIHLS and PHASE and applied. Likely changes in mortality rates for those above 60 were addressed using bootstrap estimates.
3. Education transition probabilities
  - The multi-state population model accounting for educational composition assumed that the educational transition occurred between ages 15–19. Educational transition probabilities were derived from the reference as cited [65].
4. Disability transition probabilities
  - Disability transition probabilities were estimated only for those above 60 as this paper focuses on disability among the elderly.

### Data and estimation of transition probabilities

Fertility rates, mortality rates, and the current population used to populate the model were obtained from the Department of Statistics Singapore (http://www.singstat.gov.sg) (S1–S5 Files). To estimate the disability transition probabilities, data from the two waves of the nationally representative survey of community-dwelling elderly Singaporeans was used (SIHLS & PHASE). Participants in both waves reported difficulties in any of six ADLs (taking a bath/shower, dressing up, eating, standing up from or sitting on a chair, walking around the house, and using the toilet) and seven IADLs (preparing own meals, leaving the house to purchase essentials or medication, taking care of financial matters, using the phone, dusting/cleaning up and doing other light housework, taking public transport, and taking medication as prescribed). Those who reported difficulty in performing one or more of the 13 activities on their own due to their health or physical state were considered to have functional disability. The rest, reporting no difficulties in all of the 13 activities, were considered to be in an active state. An absorbing health state, death, was included in Wave 2; information on the individual’s month and year of death was derived primarily from the Singapore Registry of Births and Deaths or secondarily from decedent interviews with next-of-kin in Wave 2.

Using the longitudinal data, the probabilities of transitioning between active and functional disability states and from active and functional disability states to death were estimated for the overall sample as well as separately for those with low education and for those with high education. For the overall sample, Eq (1) below, with age and sex as covariates, was used in the dynamic multi-state population model not accounting for educational composition. For those with low and high education respectively, Eq (2) below, with age, sex and education as covariates, was used in the dynamic multi-state population model accounting for educational composition. The equations were solved through two multinomial logistic regression models using the “multinom” function in R [66].

$$\text{ln}(\frac{p_{ij}}{p_{ii}})=\beta_{0ij}+\beta_{1ij}\cdot age+\beta_{2ij}\cdot sex$$ (1)

$$\text{ln}(\frac{p_{ij}}{p_{ii}})=\beta_{0ij}+\beta_{1ij}\cdot age+\beta_{2ij}\cdot sex+\beta_{3ij}\cdot education$$ (2)

*p*_*ij*_ is the disability transition probability from the current state ***i*** to state ***j* (*i* ≠ *j*)**, where ***i*** is active or functional disability and ***j*** is active, functional disability or death.

As suggested by the equations, the disability transition probabilities were disaggregated according to age (single age cohort from age 60—age 100+) and gender; for Eq (2), disability transition probabilities were additionally disaggregated by education.

### Probabilistic Sensitivity Analysis

After computing point estimates for the disability transition probabilities from the multinomial logistic regression models (Eqs (1) and (2) above), the bootstrap method was used to estimate the distribution of disability transition probabilities to obtain the 95% confidence interval around these point estimates. First, we standardized the sampling weights, then used the standardized weights as probabilities to draw bootstrap samples using the ‘‘sample” function in R (v3.0.2). This process was repeated 300 times. Next, disability transition probabilities were estimated using the 300 samples with the two multinomial logistic regression models. Based on the 300 sets of estimated disability transition probabilities, the distribution of age-sex-(education)-specific disability transition probabilities and 95% confidence intervals (empirical intervals) were obtained (S1–S2 Figs).

## Results

### Transition probabilities by age, gender and education

**Fig 3:**
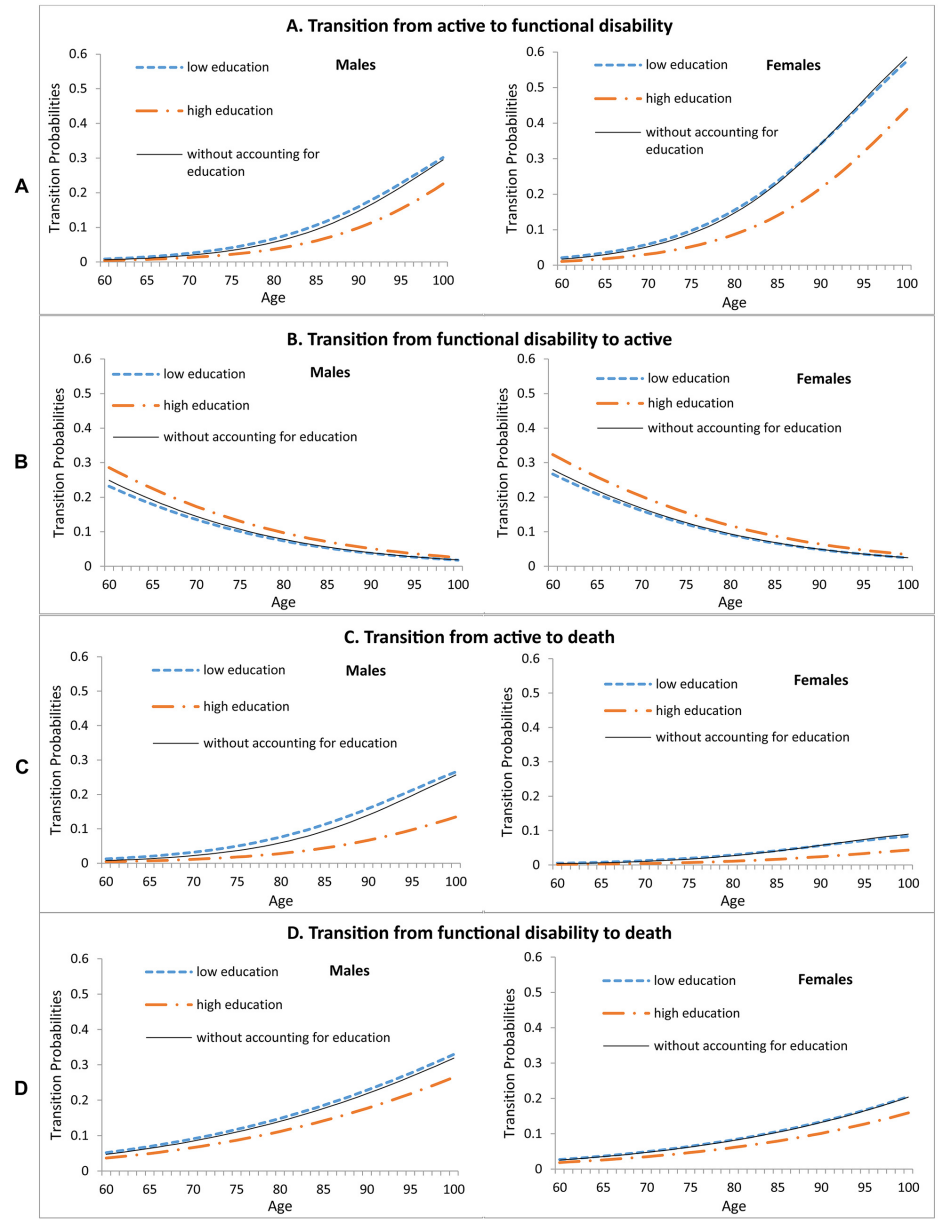
Transition Probabilities by Age, Gender and Education. (A) Transition probabilities from active to functional disability. (B) Transition probabilities from functional disability to active. (C) Transition probabilities from active to death. (D) Transition probabilities from functional disability to death.

The point estimates of the age- and gender- specific disability probabilities of transition from (a) active to functional disability (incidence of functional disability), (b) functional disability to active (recovery from functional disability), (c) active to death (mortality of active elderly) and (d) functional disability to death (mortality of elderly with functional disability), overall (solid lines) and by education (dotted / broken lines) are presented in Fig 3A–3D. For males and females at all ages, overall as well as by education, the probability of transition from active to functional disability, and from both active and functional disability states to death increased with age while recovery from functional disability decreased with age. Comparing the two education groups, for both males and females at all ages, those with low education had a higher incidence of functional disability (Fig 3A), lower recovery from functional disability (Fig 3B), and higher probability of mortality for both active (Fig 3C) and functional disability states (Fig 3D) than those with high education.

### Forecasting the number of elderly with functional disability

**Fig 4:**
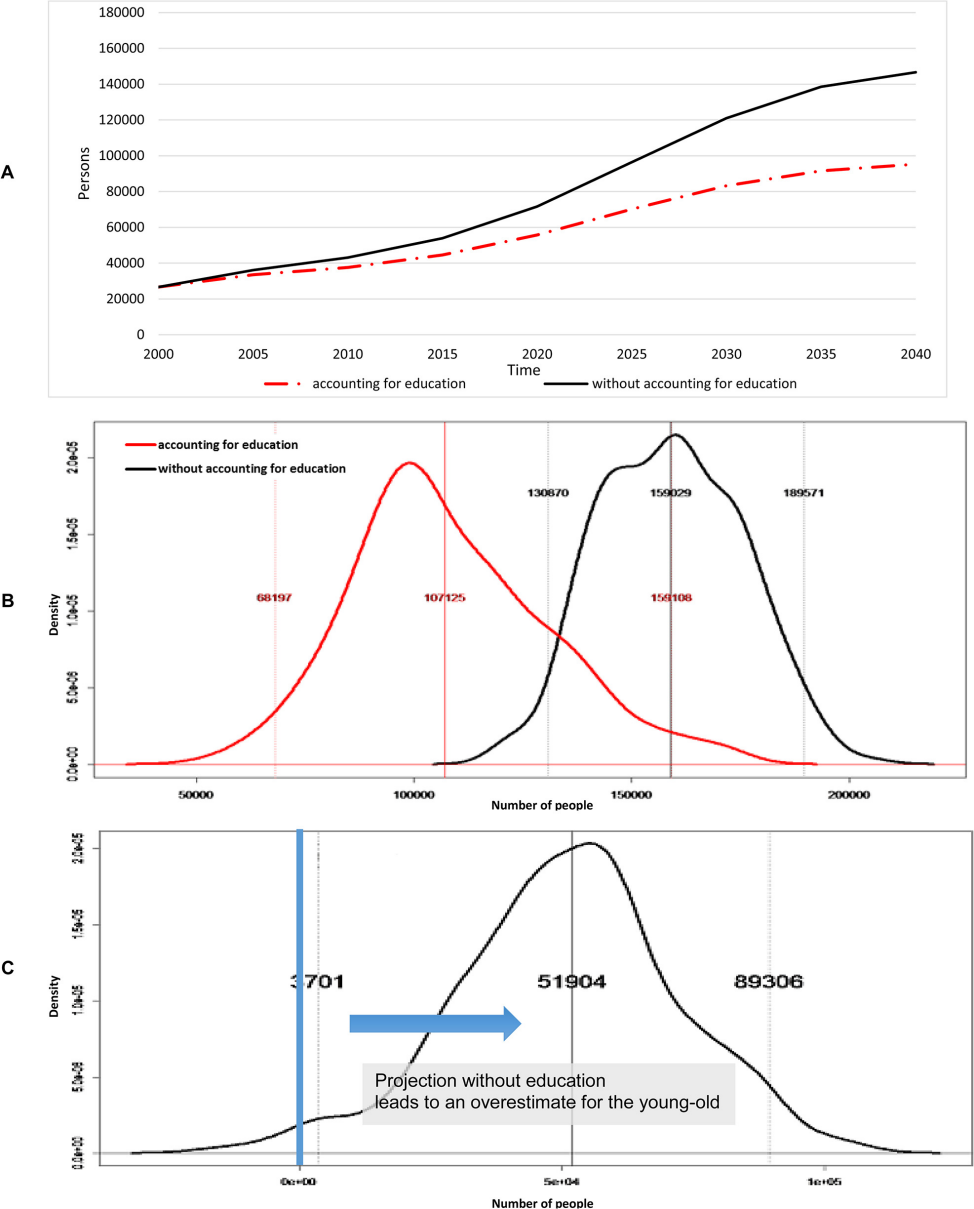
Young-old (aged 60–79). (A) Projected number of young-old with functional disability. (B) Distribution of young-old with functional disability at 2040. (C) Difference between not accounting and accounting for educational composition at 2040.

**Fig 5:**
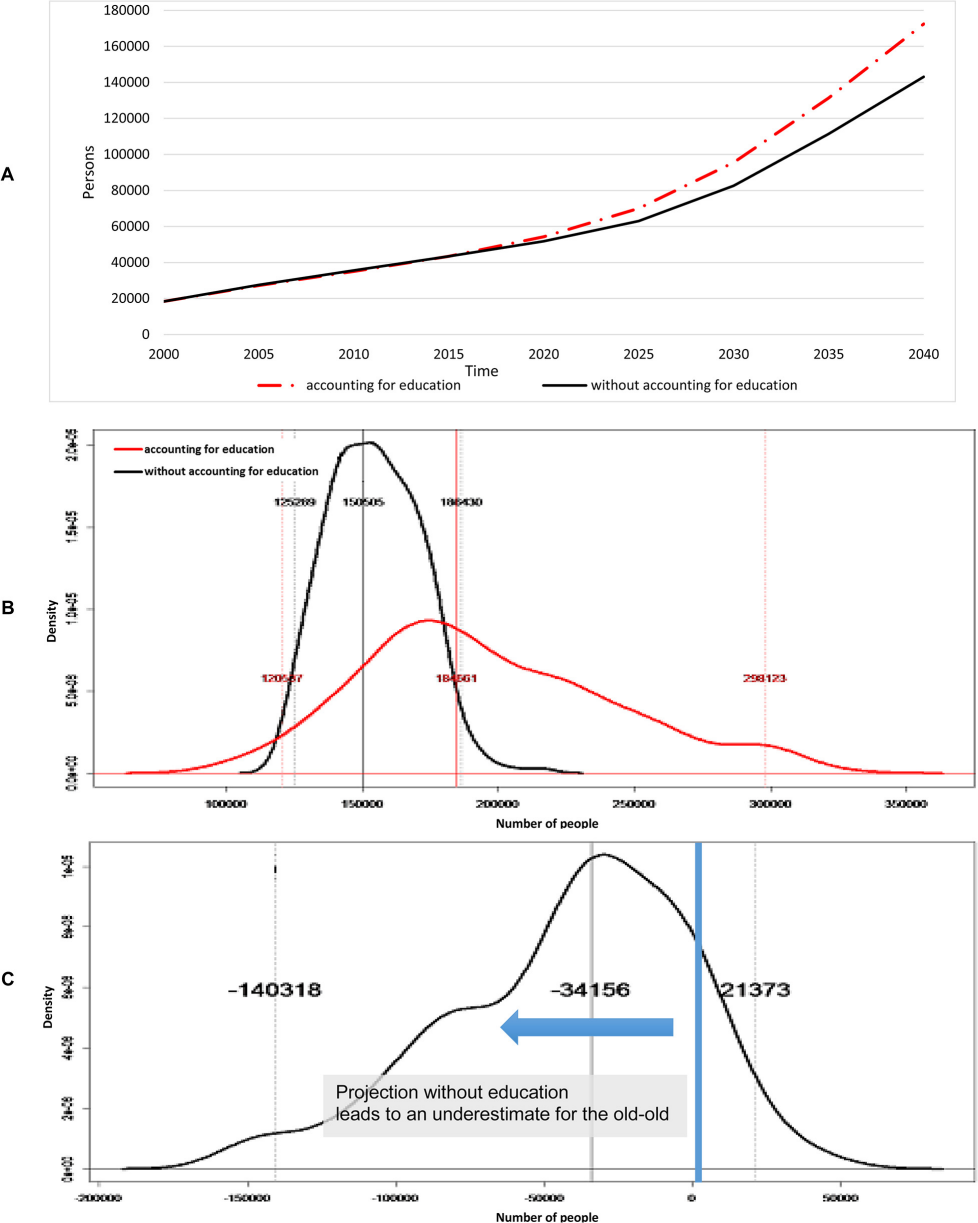
Old-old (aged 80+). (A) Projected number of old-old with functional disability. (B) Distribution of old-old with functional disability at 2040. (C) Difference between not accounting and accounting for educational composition at 2040.

We looked at the population projections for the young-old (Fig 4A) and old-old (Fig 5A) up to year 2040 and compared them to year 2000 (Table 1). Accounting for educational composition, the total elderly population was projected to increase 486 percent from about 345,800 in 2000 to approximately 1.68 million by 2040 (Table 1); of these 1.68 million elderly in 2040, approximately 95 percent were expected to have high education compared to 40 percent in 2000. Meanwhile, not accounting for educational composition, the total elderly population in Singapore was projected to increase 429% from about 345,800 in 2000 to about 1.48 million by 2040 (Table 1). Looking first at the young-old, not accounting for educational composition was projected to overestimate the young-old by 65 percent relative to the projection made accounting for educational composition (Fig 4A). Fig 4B shows the distribution of young-old with functional disability at 2040 while Fig 4C demonstrates the difference between projected young-old with functional disability with and without accounting for educational composition at 2040. These were estimated from 300 bootstrap samples, wherein transition probabilities were estimated from the same bootstrap sample. Probabilistic sensitivity analysis indicated a 98 percent likelihood that not accounting for educational composition overestimates the young-old with functional disability by 2040 (Fig 4C). The 95 percent confidence range for the effect of accounting for educational composition was an overestimation of the proportion of young-old with functional disability between 0.25 percent and 6 percent of total elderly by 2040.

**Table 1:** Projections of total elderly population, young-old with functional disability and old-old with functional disability up to the year.

Variables	Base year	Projected						% change from 2000 to 2040 (%)
	2000	2015	2020	2025	2030	2035	2040	
Total elderly population: Accounting for education	345,799	734,424	960,657	1,190,198	1,396,238	1,563,747	1,681,992	486
Total elderly population: Not accounting for education	345,799	690,297	908,267	1,130,269	1,307,214	1,434,259	1,484,558	429
Young-old with functional disability:Accounting for education	26,657	44,584	55,750	70,195	83,221	91,584	95,323	358
Young-old with functional disability: Not accounting for education	26,657	53,957	71,639	96,375	120,998	138,608	146,701	550
Old-old with functional disability: Accounting for education	18,357	43,454	54,240	70,036	95,510	131,331	172,425	939
Old-old with functional disability: Not accounting for education	18,357	43,460	51,850	62,999	82,666	111,362	143,155	780

For the old-old, not accounting for educational composition was projected to underestimate the old-old by 20 percent in the year 2040. Accounting for educational composition, the proportion of old-old with functional disability is projected to increase from 40.8 percent of the total elderly with disability in 2000 to 64.4 percent in 2040; in the meantime, not accounting for educational composition, the proportion of the old-old with functional disability is projected to increase from 40.8 percent in 2000 to 49.4 percent in 2040 (Fig 5A). Fig 5B shows the distribution of elderly with functional disability for the old-old at 2040 while Fig 5C demonstrates the difference between projected elderly individuals with functional disability with and without accounting for educational composition for the old-old at 2040. For the old-old, probabilistic sensitivity analysis suggested an 86 percent chance at 2040 (Fig 5C) that not accounting for educational composition will underestimate the old-old with functional disability; the likely underestimation was estimated to be as much as 9.4 percent of total elderly in 2040 at a 95 percent confidence level.

## Discussion

In projecting the future number of elderly with functional disability, we found that failing to account for changes in educational composition would lead to an overestimation of the young-old with functional disability and an underestimation of the old-old with functional disability. Of the elderly with functional disability, accounting for educational composition would lead to a higher projection of the proportion belonging to the old-old (64 percent by 2040) as compared to the proportion projected without accounting for educational composition (49.4 percent by 2040). Accounting for uncertainty in the transition probability estimates indicates a high probability that not accounting for educational composition will underestimate the number of old-old individuals with functional disability and overestimate the number of young-old.

These findings can be explained by the interacting effects of education on mortality and transition to and from functional disability. Higher education has three primary effects: lower mortality, lower incidence of functional disability, and higher recovery of functional disability. A lower mortality, all things equal, will increase the number of surviving elderly and shift the overall age distribution of the elderly population, with a delay, from a younger to an older elderly population. Alone, this will lead to an increase in the number of individuals with functional disability. This effect is counterbalanced by a lower incidence of functional disability and higher recovery of functional disability, which lowers the projected number of elderly with functional disability. Since the rate of transition to functional disability exceeds the rate from functional disability especially at older ages, the net effect of changing educational composition is an increase in the number with functional disability and a shift in the distribution of those with functional disability towards the old-old.

Only one study so far, to our knowledge, examines the effect of education on functional disability projections. A study by Samir and Lentzner [43] on the effect of education on adult mortality and disability used sample data from 70 countries that participated in the world health survey and reported similar results: not taking educational composition into account, under all the scenarios considered, reports a higher prevalence of disability compared to scenarios taking education into account. However, this study only projects disability prevalence for the age group 30–74 as a whole. In contrast, our study provides disability projections for the numbers of elderly with functional disability for both 60–79 year olds and 80+ year olds and compares this difference with and without accounting for educational composition. The current study is distinctive as it isolates the effect of education composition on transitions to and from functional disability, and death, and directly examines the impact of accounting for educational composition on the projections of young-old and old-old with functional disability.

In Singapore, we show that accounting for educational composition, it is highly likely that there will be fewer young-old with functional disability and more old-old with functional disability than expected without accounting for educational composition. This phenomenon should be given due consideration in policy and planning. This is potentially a cause for concern since the health profiles of the old-old differ from those of the young-old, which translates into different care needs. On average, the old-old have more ADL/IADL limitations [20], with the prevalence of ADL difficulties and ADL dependency having been shown to increase with age even after the age of 90 [24]. Similarly, the old-old are at higher risk of developing dementia than the young-old—incidence rates of dementia increase exponentially from age 65 to 90 [23] and even beyond the age of 90 [25]. In Singapore, a survey conducted by the Agency for Integrated Care supports these findings: the survey reported a higher prevalence of ADL limitations and dementia in old-old Singaporeans than in young-old Singaporeans.

First, our study’s findings will impact health care service utilization as utilization rates of both long-term and acute care services are much higher for the old-old than the young-old. Separate studies have found that the old-old population tends to have higher rates of home care use and hospitalization [20], use more ambulatory health care services (family physician visits, specialist physician visits and emergency room visits) [30], outpatient, emergency and inpatient services [36] and have a higher likelihood of visiting the emergency department, being hospitalized and staying for a longer period in the hospital [31] than the young-old cohort. The demand for long-term care services is also likely to increase as the old-old have been shown to use more post-acute care services including skilled nursing facilities and home health agency services than the young-old [32], and nursing home use has been associated with age, even among those aged 85 and above [33]. The higher risks of acquiring dementia and developing ADL/IADL limitations among the burgeoning old-old population will also affect the types of health care services demanded. Investigating the factors that affect whether an elderly individual chooses to age in place, Paganini-Hill [67] found that elderly with dementia and poor functional ability were less likely to live at home. Hence, future health care planners might consider increasing alternative living facilities or providing more comprehensive home care for the anticipated old-old group with disability and dementia to help them age in place, depending on the government’s preference for institutionalized care or home care.

The second area that will be impacted is health care expenditure. In the US, one study estimated that disability-associated health care expenditures represented 26.7% of national health spending [68], while another found that less than one-fifth of older persons (72 years or older) with disability at baseline or who developed disability during the course of the study accounted for approximately half of total health care expenditures [69]. Dementia also places a substantial financial burden on individuals. Each patient above 40 years of age with dementia in Indiana spent almost $10,000 more per year than each patient without dementia (largely due to more days spent in nursing homes) and the total expenditure of the cohort with dementia was 1.78 times that of the cohort without dementia; when analyzed within specific age stratum, dementia-associated expenditures accounted for 15% and 17% of total health expenditures of patients aged 65+ and 80+ respectively [70]. Moreover, numerous studies have shown that aging populations are associated with greater health care expenditure [27–29, 32, 71]. All of these studies indicate escalating costs due not only to an aging population, but also due to a large old-old cohort who suffer from dementia and disability limitations, which will be of great concern to policymakers.

The third implication of a much larger old-old population with disability and dementia is an increase in care needs and consequently an increased demand for both formal and informal caregiving. A smaller proportion of the old-old group is likely to have a spouse to provide care; hence caregiving duties are likely to be taken on by their children, many of whom will belong to the young-old group, foreign domestic workers, or formal home and institutional care service providers. This could affect labor participation as studies have shown that some informal caregivers of elderly with functional disability choose to withdraw from the labor force to cope with the demands of caregiving [39, 72]. Furthermore, caregivers are also thought to use more health care services [73] and have a higher chance of developing depression [74]. Programs that tap into the larger active young-old group (60–79 years old) to care for the population of elderly with functional disability could potentially alleviate the high demand for caregiving. In countries like the United States, programs have been implemented where active elderly people volunteer to help their elderly neighbors with daily activities like shopping and doing housework as well as regularly check in with them and refer them to professional services when necessary [75]. Private companies have also hired seniors to act as caregivers for other seniors in need [76]. These creative programs benefit the active elderly by providing them with some income and may be a source of meaningful engagement for them. At the same time, such programs enable informal caregivers who might have left their jobs to care for elderly with functional disability to continue working. As demands for caregiving increase, such programs will become even more important in reducing the care burden of employed caregivers.

Another point worth considering is making provisions for the larger pool of healthy active young-old to continue working in order to increase elderly participation in the labor market. In Singapore, there has been ongoing discussion about extending the statutory retirement age, currently 62, to 65, or even to 70, in order to cope with a local labor shortage [77]. While some might argue that such a move would be politically unpopular, a 2003 study examining the attitudes of older workers towards work and retirement in Singapore suggested that work was a significant part of elderly Singaporeans’ lives and that they would prefer to remain partially employed even after reaching the official retirement age [78]. One caveat is the gap between people’s expressed intentions and ultimate actions. Regardless, the active elderly population is a potential resource the Singaporean government can tap into. Currently, the Singaporean government adopts policies such as allowing employers to re-evaluate the seniority-based wage system for employees above the age of 62 in order to make it cost-effective to hire older workers [79]. At the same time, the government supports elderly workers through job re-training and upgrading and workplace redesign. Continuing to create incentives for elderly people to work while negotiating with and encouraging employers to provide good working terms for their elderly employees will help to ease the long-run labor shortage.

The dynamic multi-state population models presented have three limitations. First, the number of elderly with functional disability relies largely on the projected population trend in Singapore. Any changes observed in the population trend, especially due to immigration policies, are likely to change the numerical values observed in the simulation. Second, the broad aggregation of education into low and high educational levels due to an inadequate survey sample in estimating the transition probabilities is likely to lessen the full impact of education on functional disability. This may lead to an over or under estimation of the number of elderly with functional disability. Third, given the relatively short interval between the two waves of the survey, there were a limited number of transitions that occurred during that time period; hence, our sample size was too small to disaggregate functional disability into further sub-categories.

## Conclusion

This paper reinforces the significance of considering educational composition in projections of the number of people with functional disability, and in this context has illustrated the importance of differentiating the complex dynamic effects of disability onset and recovery on the young-old and the old-old. In the case of Singapore, the most salient impact of the changing educational composition of the elderly population will be a small population of young-old with functional disability and a larger population of old-old with functional disability. With this in mind, policymakers, especially in countries like China, South Korea, Thailand and Singapore where future elderly are projected to be significantly more educated, should be aware of the potential value in further tracking the relationship between socio-economic factors such as education and needs related to functional disability. Policymakers would also benefit from a proactive approach that considers the effect of education on future disability and demographic changes when planning healthcare services and designing labor market polices.

## Supporting Information

S1 Fig Probabilistic Sensitivity Analysis Result from Bootstrap for Young-old with Functional Disability. (A) Accounting for educational composition. (B) Not accounting for educational composition.
(TIF)

S2 Fig Probabilistic Sensitivity Analysis Result from Bootstrap for Old-old with Functional Disability. (A) Accounting for educational composition. (B) Not accounting for educational composition.
(TIF)

S3 Fig Projected Total Elderly Population. (TIF)

S1 File Abridged Life Tables of Singapore, 1990–2002. (XLS)

S2 File Complete Life Tables of Singapore, 2003–2012. (XLS)

S3 File Population Trends of Singapore, 2013. (XLS)

S4 File Basic Demographic Characteristics of Singapore, 2000. (XLS)

S5 File Population Education Profile of Singapore, 2000. (XLS)
