# Supplementary figures and images for: Projection of Young-Old and Old-Old with Functional Disability: Does Accounting for the Changing Educational Composition of the Elderly Population Make a Difference?
Source: PLoS One. 2015 May 14;10(5):e0126471. doi: 10.1371/journal.pone.0126471 (PMC4431717; doi:10.1371/journal.pone.0126471)

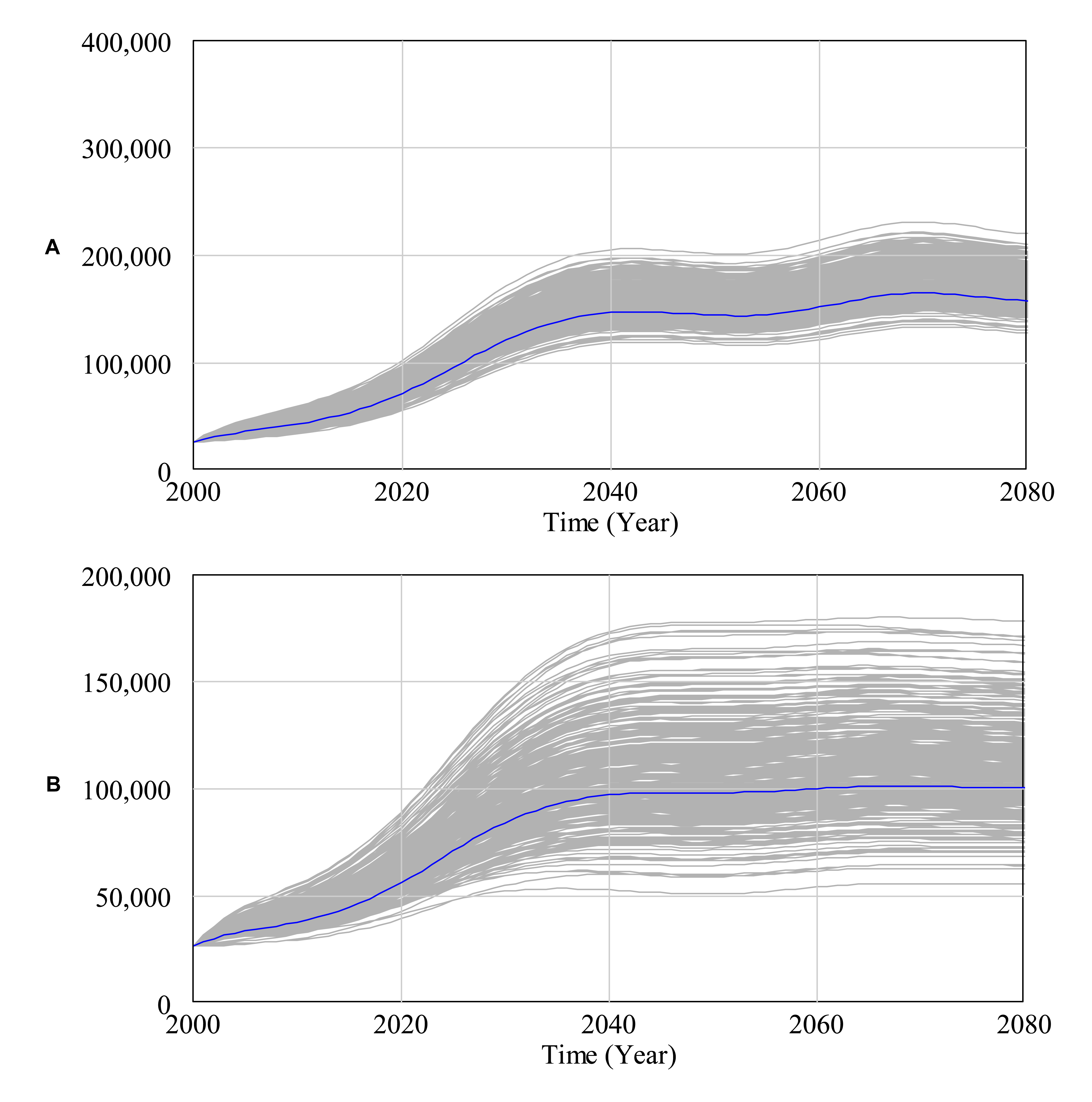

Supplement: S1 Fig — (A) Accounting for educational composition. (B) Not accounting for educational composition. (TIF) [file pone.0126471.s001.tif]

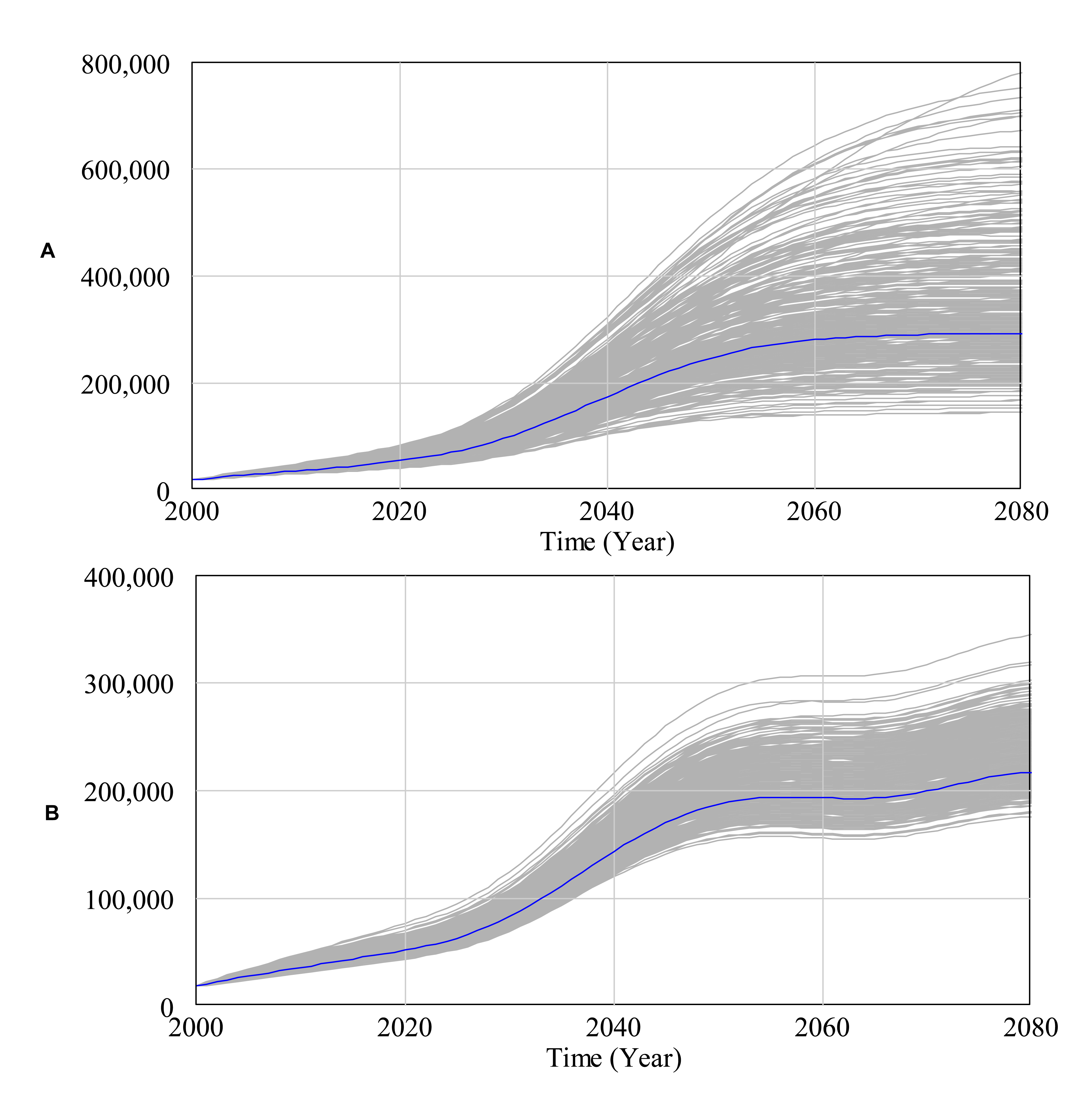

Supplement: S2 Fig — (A) Accounting for educational composition. (B) Not accounting for educational composition. (TIF) [file pone.0126471.s002.tif]

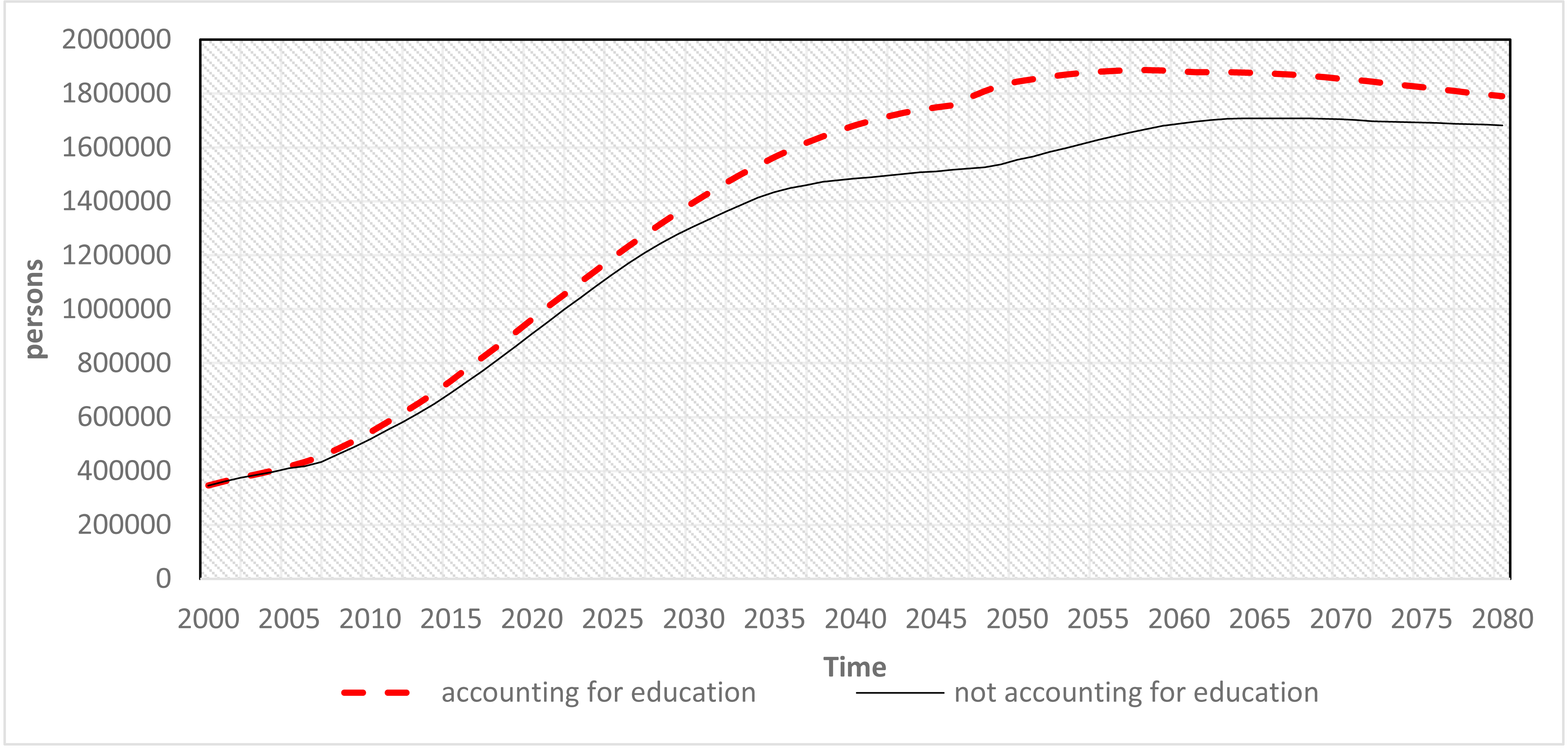

Supplement: S3 Fig — (TIF) [file pone.0126471.s003.tif]
